# Understanding the Genetics of Early-Onset Obesity in a Cohort of Children From Qatar

**DOI:** 10.1210/clinem/dgad366

**Published:** 2023-06-17

**Authors:** Idris Mohammed, Basma Haris, Tara Al-Barazenji, Dhanya Vasudeva, Sara Tomei, Iman Al Azwani, Hajar Dauleh, Saira Shehzad, Shiga Chirayath, Ghassan Mohamadsalih, Goran Petrovski, Amel Khalifa, Donald R Love, Mashael Al-Shafai, Khalid Hussain

**Affiliations:** College of Health & Life Sciences, Hamad Bin Khalifa University, PO Box 34110, Doha, Qatar; Division of Endocrinology, Department of Pediatric Medicine, Sidra Medicine, PO Box 26999, Doha, Qatar; Division of Endocrinology, Department of Pediatric Medicine, Sidra Medicine, PO Box 26999, Doha, Qatar; Department of Biomedical Sciences, College of Health Sciences, QU Health, Qatar University, PO Box 2713, Doha, Qatar; Division of Endocrinology, Department of Pediatric Medicine, Sidra Medicine, PO Box 26999, Doha, Qatar; Omics Core, Integrated Genomic Services, Research Branch, Sidra Medicine, PO Box 26999, Doha, Qatar; Omics Core, Integrated Genomic Services, Research Branch, Sidra Medicine, PO Box 26999, Doha, Qatar; Division of Endocrinology, Department of Pediatric Medicine, Sidra Medicine, PO Box 26999, Doha, Qatar; Division of Endocrinology, Department of Pediatric Medicine, Sidra Medicine, PO Box 26999, Doha, Qatar; Division of Endocrinology, Department of Pediatric Medicine, Sidra Medicine, PO Box 26999, Doha, Qatar; Division of Endocrinology, Department of Pediatric Medicine, Sidra Medicine, PO Box 26999, Doha, Qatar; Division of Endocrinology, Department of Pediatric Medicine, Sidra Medicine, PO Box 26999, Doha, Qatar; Division of Endocrinology, Department of Pediatric Medicine, Sidra Medicine, PO Box 26999, Doha, Qatar; Division of Genetic Pathology, Department of Pathology, Sidra Medicine, PO Box 26999, Doha, Qatar; Department of Biomedical Sciences, College of Health Sciences, QU Health, Qatar University, PO Box 2713, Doha, Qatar; Biomedical Research Center, Qatar University, PO Box 2713, Doha, Qatar; Division of Endocrinology, Department of Pediatric Medicine, Sidra Medicine, PO Box 26999, Doha, Qatar

**Keywords:** monogenic obesity, severe obesity, childhood obesity, *MC4R*, Qatar

## Abstract

**Context:**

Monogenic obesity is a rare form of obesity due to pathogenic variants in genes implicated in the leptin–melanocortin signaling pathway and accounts for around 5% of severe early-onset obesity. Mutations in the genes encoding the MC4R, leptin, and leptin receptor are commonly reported in various populations to cause monogenic obesity. Determining the genetic cause has important clinical benefits as novel therapeutic interventions are now available for some forms of monogenic obesity.

**Objective:**

To unravel the genetic causes of early-onset obesity in the population of Qatar.

**Methods:**

In total, 243 patients with early-onset obesity (above the 95% percentile) and age of onset below 10 years were screened for monogenic obesity variants using a targeted gene panel, consisting of 52 obesity-related genes.

**Results:**

Thirty rare variants potentially associated with obesity were identified in 36 of 243 (14.8%) probands in 15 candidate genes (*LEP*, *LEPR*, *POMC*, *MC3R*, *MC4R*, *MRAP2*, *SH2B1*, *BDNF*, *NTRK2*, *DYRK1B*, *SIM1*, *GNAS*, *ADCY3*, *RAI1*, and *BBS2*). Twenty-three of the variants identified were novel to this study and the rest, 7 variants, were previously reported in literature. Variants in *MC4R* were the most common cause of obesity in our cohort (19%) and the c.485C>T p.T162I variant was the most frequent *MC4R* variant seen in 5 patients.

**Conclusion:**

We identified likely pathogenic/pathogenic variants that seem to explain the phenotype of around 14.8% of our cases. Variants in the *MC4R* gene are the commonest cause of early-onset obesity in our population. Our study represents the largest monogenic obesity cohort in the Middle East and revealed novel obesity variants in this understudied population. Functional studies will be required to elucidate the molecular mechanism of their pathogenicity.

Obesity results from a disequilibrium between energy intake and energy expenditure, leading to excessive body fat storage as triglycerides in white adipocytes. Obesity is the main cause of several chronic and preventable diseases such as type 2 diabetes and insulin resistance, fatty liver, dyslipidemia, hypertension, cardiovascular diseases, and several cancers (1, 2).

Genetic causes of obesity are classified into 2 broad groups: monogenic and polygenic. Of these 2, monogenic obesity accounts for only 5% of obesity in outbred populations (3). Polygenic obesity, also known as common obesity, resembles complex diseases in terms of heritability, in which several single nucleotide polymorphisms (SNPs), gene–gene interactions, or gene–environment interactions play roles in its pathogenicity. Monogenic obesity usually presents in early childhood and is due to a single gene disease-causing variant(s), mainly genes implicated in the leptin–melanocortin pathway, leading to defects in the regulation of hunger and satiety in the hypothalamus. Unlike common obesity, monogenic obesity is rare and is characterized by severe, early-onset obesity (usually appearing before the age of 10 years) (4).

Following the completion of the human genome project, next-generation sequencing (NGS) holds the promise to transform the field of genetics and may benefit patients by enabling genetic-aided therapeutic approaches. NGS has facilitated the discovery of the genetic causes of obesity in various population using genome-wide association studies (5). Even though several studies have investigated monogenic obesity genes at a small scale, some studies have screened for variants in obesity-associated genes using comprehensive targeted gene panels. A Norwegian cohort which looked into *LEP*, *LEPR*, *MC4R*, *PCSK1*, and *POMC* found 4 cases (0.8%) pathogenic or likely pathogenic *MC4R* variant (6). In a Turkish study of 105 children with early-onset obesity that screened 41 monogenic obesity genes, around 10.5% of the cases were found to carry genetic variants in the *SIM1*, *POMC*, *PSCK1*, *MC4R*, and *LEPR* genes (7).

In a study of 25 obese Guadeloupean patients, 5 heterozygous variants in 4 monogenic obesity genes (*MC4R*, *NTRK2*, *SH2B1*, and *SIM1*) were detected with a prevalence of 10% (8). A cohort of 209 obese patients from Italy which screened *MC4R*, *LEP*, and *LEPR* genes detected only 1 novel *MC4R* frameshift variant that impairs *MC4R* signaling (9).

The highest prevalence of monogenic obesity was seen in a highly consanguineous population of Pakistan in a study of obese children that investigated only 3 monogenic obesity genes (*LEP*, *LEPR*, and *MC4R)* and found around 30% of the cases carried pathogenic variants (10).

According to a recently published systematic review, the prevalence of obesity and overweight in the Middle East is estimated to be 33.14% and 21.17%, respectively (11). The prevalence of overweight and obesity among school children and adolescents aged 5-19 years in Qatar is 44.8% and 40.4%, respectively, and in a subgroup of children aged 5-9 years it is 18.3% and 18.2%, respectively (12). A recently published systematic review reported thirty monogenic obesity cases from the Arab world (13).

Despite the high rates of obesity including childhood obesity, the genetics of obesity is largely understudied in this highly consanguineous Middle Eastern population. Identifying the genetic causes of monogenic obesity especially during childhood has a great clinical benefit for the patients in relation to the disease prognostics, personalized therapeutic interventions, management of obesity-associated complications and genetic counseling for the family members. Here, we investigated the genetic landscape of monogenic obesity among children (age of onset less than 10 years) in a large cohort of 243 children with severe early-onset obesity from Qatar.

## Materials and Methods

**Figure 1.**
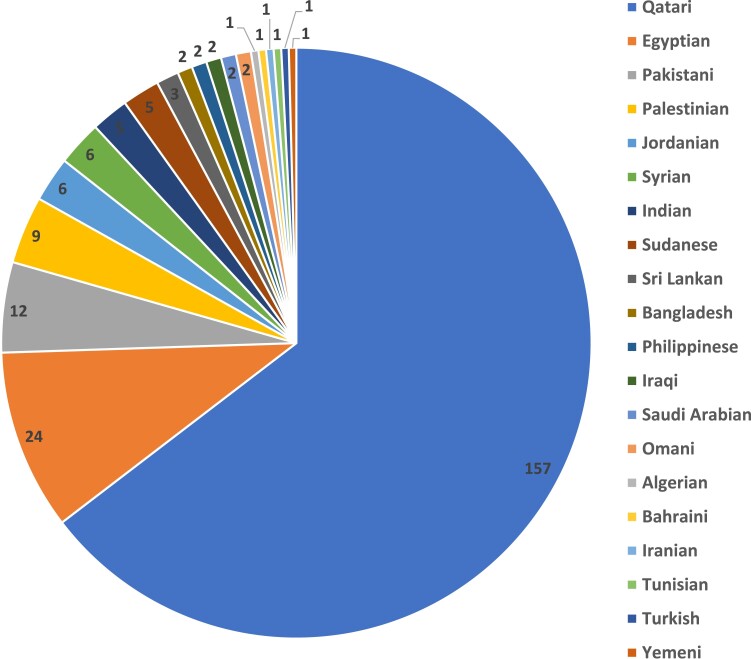
Geographical distribution of patients who met the inclusion criteria of the cohort. The majority of the patients (157/243) are Qataris, the rest (86/243) are patients residing in Qatar with Middle Eastern background.

The participants of this study comprised 243 patients with a main phenotype of severe early-onset obesity (age of onset: 3 months to 10 years) who were seen in the endocrinology clinic at Sidra Medicine, Doha, Qatar, between 2018 and 2022. All participants met the inclusion criteria of body mass index (BMI), percentile (greater or equal to the 95% percentile), and age of onset (10 years or below) (Fig. 1). The height and weight of all participants were measured in the clinic and BMI percentile was calculated using the World Health Organization child growth standards software (World Health Organization Anthro version 3.2.2 and AnthroPlus) (Table 1). Peripheral blood was collected in EDTA tubes for DNA extraction from probands following signed assent and consent for parental permission. The study was approved by the Institutional Review Board Institutional Review Board for the protection of human subjects in Sidra Medicine, Qatar (reference number 1689931).

**Table 1.** Clinical characterizations of the patients with rare variants in monogenic obesity genes

Case	Gene	Variant	Origin	Sex	Age at diagnosis (years)	Age of onset (years)	Birth weight (kg)	Weight (kg)	Centile	BMI (kg/m^2^)	Associated phenotypes
P1	*MC4R*	p.C271R	Qatari	F	9	0.25	4.7	128	100	48.6	Hyperphagia, fatty liver, OSA, AN
P2	*MC4R*	p.I170V	Qatari	F	4	0.7	3	27	99	27.8	Hyperphagia
P3	*MC4R*	p.S85G, p.Y268H	Jordanian	F	8	0.25	3.5	65	99	36.4	Hyperphagia
P4	*MC4R*	p.T162I	Qatari	M	2	0.5	2.5	44	100	29.1	Hyperphagia, Asthma, OSA
P5	*MC4R*	p.T162I	Qatari	M	2	1	3.8	29	100	28.7	Hyperphagia, fatty liver, hepatomegaly, OSA, hyperactivity
P6	*MC4R*	p.T162I	Qatari	F	4	2	2.8	64	100	39.4	Hyperphagia, mild OSA
P7	*MC4R*	p.T162I	Qatari	M	12	0.5	3.7	188	100	69	fatty liver, hepatomegaly, Snoring
P8	*MC4R*	p.T162I	Qatari	M	1	6	2.5	20	NA	26	Snoring, macrocephaly
P9	*MC4R*	p.I69R	Omani	M	5	3	3	64	100	41	Hyperphagia, polyuria and polydipsia, insulin resistance, AN
P10	*MC4R*	p.I69R	Omani	F	3	1	2.9	31	100	39	Hyperphagia, polyuria, AN, dyslipidemia
P11	*LEPR*	p.S961T	Palestinian	M	15	4	NA	112	100	33	Insulin resistance, fatty parenchyma of the pancreas
P12	*LEPR*	p.N826S p.H902R	Saudi	F	2	1	NA	33	99	28	Hyperphagia, AN, mild OSA
P13	*LEPR*	p.N826S p.H902R	Qatari	M	2	1	2.7	29	97	20	Mild DD, snoring
P14	*LEPR*	p.N826S p.H902R	Qatari	F	2	1	NA	26	97	24	Hyperphagia
P15	*LEPR*	p.N826S p.H902R	Qatari	F	3	2	3.5	28	100	27	Macrocephaly, thyroid abnormalities
P16	*LEP*	c.-29+1G>A*^a^*	Egyptian	F	5	0.5	3.4	78	100	48	Hyperphagia, OSA, fatty liver
P17	*LEP*	c.-29+1G>A*^a^*	Egyptian	F	8	2	3	98	100	44.2	Hyperphagia, OSA, fatty liver
P18	*POMC*	p.F82V	Indonesian	M	13	10	3.1	122	100	44	Insulin resistance, AN
P19	*POMC*	p.K76Q	Qatari	F	2	1	3.9	24	99	25	—
P20	*ADCY3*	p.G408R	Egyptian	F	17	5	NA	111	100	44	Heterogeneous thyroid gland
P21	*ADCY3*	p.G408R	Qatari	M	12	6	3.8	190	99	67	Fatty liver and pancreas, prediabetes, severe AN
P22	*ADCY3*	p.T840X	Pakistani	F	2	0.75	3.5	26	100	27	Mild hepatomegaly, insulin resistance
P23	*MRAP2*	p.E157G	Egyptian	M	4	2	NA	40	100	34	Hypothyroidism hepatomegaly fatty liver and pancreas
P24	*SIM1*	p.S663L	Qatari	M	14	10	3.5	99.6	99	36	—
P25	*SIM1*	p.H143Pfs*18	Qatari	F	11	4	NA	2.96	100	48	hepatomegaly, hepatic steatosis, autoimmune thyroiditis, prediabetes, facial dysmorphism
P26	*NTRK2*	p.L576F	Qatari	M	4	2	NA	22.2	100	26	Hyperphagia
P27	*BDNF*	R298Q	Qatari	M	6	3	3	43.9	100	34	Hepatomegaly, Hyperactivity, hyperphagia
P28	*BDNF*	L199P	Qatari	M	3	birth	3	32	100	31	Hyperphagia
P29	*SH2B1*	p.P640L	Qatari	M	14	6	NA	149	100	47	Hepatomegaly, splenomegaly
P30	*SH2B1*	p.P22Q, p.G269V	Palestinian	F	4	2	3.2	38	99	30	Hyperphagia, Hepatomegaly, splenomegaly, fatty liver, asthma, subclinical hypothyroidism
P31	*SH2B1*	p.V756M	Sudanese	M	8	3	3.8	59	99	30	Hyperphagia, T2D, insulin resistance
P32	*SH2B1*	p.V756A	Egyptian	M	7	5	5	67	99	31	Hyperphagia
P33	*DYRK1B*	p.P508S	Bangladeshi	M	4	3	2.6	118	100	35	T2D, hypertension, Fatty liver with hepatomegaly. insulin resistance
P34	*MC3R*	p.H70R	Qatari	M	17	10	3	110	100	37	—
P35	*MC3R*	p.R215W	Sudanese	M	3	1	3.5	32	100	23	Fatty liver, OSA
P36	*GNAS*	p.R228C	Indian	M	1	1	NA	23	NA	31	—
P37	*RAI1*	p.R262Q	Egyptian	M	5	1	4	37	99	26	Hyperphagia, behavioral abnormalities
P38	*BBS2*	p.Y317C	Somali	M	4	2	NA	32	100	28	developmental delay, polydactyly, hypothyroidism

Abbreviations: AN, acanthosis nigricans; NA, not available; OSA, obstructive sleep apnea.

^a^Indicates homozygous variant.

### DNA Extraction and Exome Sequencing

Genomic DNA was extracted from the peripheral leukocytes of each proband using QIAamp DNA Blood Midi Kit (Cat. 51185, Qiagen, Germany) according to the manufacturer's protocol. Genomic DNA concentrations and purities were assessed using Nanodrop 2000 spectrophotometer (Thermoscientific, Waltham, MA, USA). For NGS, exonic regions of all genes of interest were captured using an optimized set of DNA hybridization probes. The captured DNA was sequenced by massively parallel sequencing using the Illumina NovaSeq 6000 platform reversible dye terminator (Illumina, San Diego, CA, USA). Alternatively, the polymerase chain reaction (PCR) was used to amplify regions of insufficient coverage by NGS sequencing, these regions were amplified separately and resequenced. We targeted regions that included all the exons and intron–exon boundaries of 52 genes, which have roles in energy homeostasis and adipose tissue proliferation (*ADCY3*, *ALMS1*, *ARL6*, *BBIP1*, *BBS1*, *BBS10*, *BBS12*, *BBS2*, *BBS4*, *BBS5*, *BBS7*, *BBS9*, *BDNF*, *CEP290*, *CFAP418*, *CPE*, *CUL4B*, *DYRK1B*, *GNAS*, *IFT172*, *IFT27*, *IFT74*, *KSR2*, *LEP*, *LEPR*, *LZTFL1*, *MAGEL2*, *MC3R*, *MC4R*, *MCHR1*, *MKKS*, *MKS1*, *MRAP2*, *NCOA1*, *NR0B2*, *NTRK2*, *PCSK1*, *PHF6*, *POMC*, *PPARG*, *RAB23*, *RAI1*, *SDCCAG8*, *SH2B1*, *SIM1*, *TMEM67*, *TRIM32*, *TTC8*, *TUB*, *UCP3*, *VPS13B*, *WDPCP*). A summary of the genes in the panel is presented in (Table 2). PCR products were purified using the Qiaquick PCR Purification Kit (Qiagen, cat. no. 28106). Cycle sequencing was carried out using an ABI Big Dye terminator v3.1. kit, and products were resolved by electrophoresis in an ABI 3730 capillary Genetic Analyzer (Thermo Fisher Scientific, Waltham, MA, USA) according to the manufacturer's protocol. The gene panel provides 99.9% coverage of the relevant coding sequences including approximately 10 base pair of flanking sequence. The binary base call files were converted to FASTQ by bcl2fastq v.2.20. The FASTQ files containing quality score and nucleotide sequence were aligned to the human reference genome GRCh37/hg19 using Burrows–Wheeler Aligner (BWA v.0.7.17). Sequence alignment map format (SAM) was converted to binary alignment map (BAM) format using Samtools package. The resulting BAM files were then realigned using the Genome Analysis kit (GATK v.3.7) for discovery of SNPs and small insertions and deletions. Finally, variants were annotated using SNPEff with respect to their impact to protein-coding genes in the ENSEMBL database. The following quality metrices were achieved: the targeted sequencing panel coverage reached up to 700 times in some cases and a mean coverage greater than 300 times (>99th percentile of the bases were covered). Sanger sequencing was performed in both the forward and reverse directions for confirmation of the variants identified.

**Table 2.** List of the 52 genes included in the targeted panel

No.	Gene symbol	Gene name	Reference sequence	OMIM ID	Chromosomal location	Inheritance
1	***ADCY3***	Adenylate cyclase 3	NM_004036	600291	2p23.3	AR
2	*ALMS1*	Alms1 centrosome and basal body associated protein	NM_015120.4	606844	2p13.3	AR
3	*BBS1*	Bardet–Biedl syndrome 1	NM_024649.4	209901	11q13.3	AR
4	*BBS2*	Bardet–Biedl syndrome 2	NM_031885.3	606151	16q13	AR
5	*BBS3 (ARL6)*	Bardet–Biedl syndrome 3	NM_032146.5	608845	3q11.2	AR
6	*BBS4*	Bardet–Biedl syndrome 4	NM_033028.4	600374	15q24.1	AR
7	*BBS5*	Bardet–Biedl syndrome 5	NM_152384.2	603650	2q31.1	AR
8	*BBS6 (MKKS)*	Bardet–Biedl syndrome 6	NM_018848.3	604896	20p12.2	AR
9	*BBS7*	Bardet–Biedl syndrome 7	NM_176824.2	607590	4q27	AR
10	*BBS8 (TTC8)*	Bardet–Biedl syndrome 8	NM_001288781.1	608132	14q31.3	AR
11	*BBS9*	Bardet–Biedl syndrome 9	NM_198428.2	60968	7q14.3	AR
12	*BBS10*	Bardet–Biedl syndrome 10	NM_024685.3	610148	12q21.2	AR
13	*BBS11 (TRIM32)*	Bardet–Biedl syndrome 11	NM_012210.3	602290	9q33.1	AR
14	*BBS12*	Bardet–Biedl syndrome 12	NM_152618.2	610683	4q27	AR
15	*BBS13 (MKS1)*	Bardet–Biedl syndrome 13	NM_017777.3	609883	17q22	AR
16	*BBS14 (CEP290)*	Bardet-Biedl syndrome 14	NM_025114.3	610142	12q21.32	AR
17	*BBS14 (TMEME67)*	Bardet–Biedl syndrome 14	NM_153704.5	609884	8Q22.1	AR
18	*BBS15 (WDPCP)*	Bardet-Biedl syndrome 15	NM_015910.5	613580	2p15	AR
19	*BBS16 (SDCCAG8)*	Bardet-Biedl syndrome 16	NM_006642.3	613524	1q43-44q	AR
20	*BBS17 (LZTFL1)*	Bardet–Biedl syndrome 17	NM_020347.3	606568	3q21.31	AR
21	*BBS18 (BBIP1)*	Bardet–Biedl syndrome 18	NM_001195304.1	613605	10q25.2	AR
22	*BBS19 (IFT27)*	Bardet–Biedl syndrome 19	NM_006860.4	615870	22q12.3	AR
23	*BBS20 (IFT172)*	Bardet–Biedl syndrome 20	NM_015662.2	607386	2p23.1	AR
24	*BBS21 (CFAP418)*	Bardet–Biedl syndrome 21	NM 177965.3	614477	8q22.1	AR
25	*BBS22 (IFT74)*	Bardet–Biedl syndrome 22	NM_025103.2	608040	9q21.2	AR
26	***BDNF***	Brain-derived neurotrophic factor	NM_001143810.1	113505	11p14.1	AD
27	*CPE*	Carboxypeptidase E	NM_001873.3	114855	4q32.3	AR
28	*CUL4B*	Cullin 4B	NM_003588.3	300304	Xq24	XLR
29	*DYRK1B*	Dual-specificity tyrosine phosphorylation regulated kinase 1B	NM_004714.2	604556	19q13.2	AD
30	*GNAS*	Guanine nucleotide binding protein alpha stimulating	NM_000516.5	139320	20q13.32	AD
31	*KSR2*	Kinase suppressor of RAS 2	NM_173598.4	610737	12q24.2	AD
32	***LEP***	Leptin	NM_000230.2	164160	7q32.1	AR
33	***LEPR***	Leptin receptor	NM_002303.5	601007	1p31.3	AR
34	*MAGEL2*	Mage-like 2	NM_019066.4	605283	15q11.2	AD
35	***MC3R***	Melanocortin 3 receptor	NM_019888.3	155540	20q13.2	AD, UK
36	***MC4R***	Melanocortin 4 receptor	NM_005912.2	155541	18q21.32	AD, AR
37	*MCHR1*	Melanin concentrating hormone receptor 1	NM_005297.3	601751	22q13.2	AR, UK
38	*MRAP2*	Melanocortin 2 receptor accessory protein 2	NM_138409.3	615410	6q14.2	AD
39	*NCOA1*	Nuclear receptor coactivator 1	NM 003743.4	602691	2q23.3	AD
40	*NR0B2*	Nuclear receptor subfamily 0 group B member 2	NM_021969.2	604640	Xp21.2	AD, AR, MU
41	*NTRK2*	Neurotrophic receptor tyrosine kinase 2	NM_006180.4	600456	9q21.3	AD
42	*PCSK1*	Proprotein convertase subtilisin kexin type 1	NM_001177875.1	162150	5q15	AR, UK
43	*PHF6*	PHD finger protein 6	NM_032458.2	300414	Xq26.2	XLR
44	*POMC*	Proopiomelanocortin	NM_001035256	176830	2p23.3	AD, AR, UK
45	*PPARG*	Peroxisome proliferator activated receptor gamma	NM_015869.4	601487	3p25.2	AD, AR, UK
46	*RAB23*	Ras associated protein 23	NM_183227.2	606144	6p12.1	AR
47	*RAI1*	Retinoic acid induced 1	NM_030665.3	607642	17p11.2	AD, IC
48	*SH2B1*	Src homology 2B 1	NM_001145795.1	608937	16p11.2	AD
49	*SIM1*	Single-minded homolog 1	NM_005068.2	603128	6q16.3	AD
50	*Tub*	Tub bipartite transcription factor	NM_003320.4	601197	11p15.4	AR
51	*UCP3*	Uncoupled protein 3	NM_003356.3	602044	11q13.4	AD, AR. MU
52	*VPS13B*	Vacuolar protein sorting 13 homolog B	NM_017890.4	607817	8q22.2	AR

Genes in bold indicate a well-established link with monogenic obesity.

Abbreviations: AD, autosomal dominant; AR, autosomal recessive; IC, isolated cases; MU, multifactorial; UK, unknown; XLR, X-linked recessive.

### Sequencing Data Analysis

Human Genome Variation Society recommendations were followed to define sequence variants (http://www.hgvs.org). Variants different from the reference genome (GRCh37, hg19) were categorized into 5 classes: pathogenic, likely pathogenic, variant of uncertain significance, likely benign, and benign according to the standards and guidelines of the American College of Medical Genetics and Genomics (ACMG) (14). The function of the genetic variants detected in this study were examined using in silico prediction tools such as Sorting Intolerant From Tolerant (SIFT https://sift.bii.a-star.edu.sg/), Polymorphism Phenotyping v2 (Polyphen-2 http://genetics.bwh.harvard.edu/pph2/), MutationTaster (http://www.mutationtaster.org/), Combined Annotation Dependent Depletion (CADD https://cadd.gs.washington.edu/), and winterVar (https://wintervar.wglab.org/).

## Results

**Figure 2.**
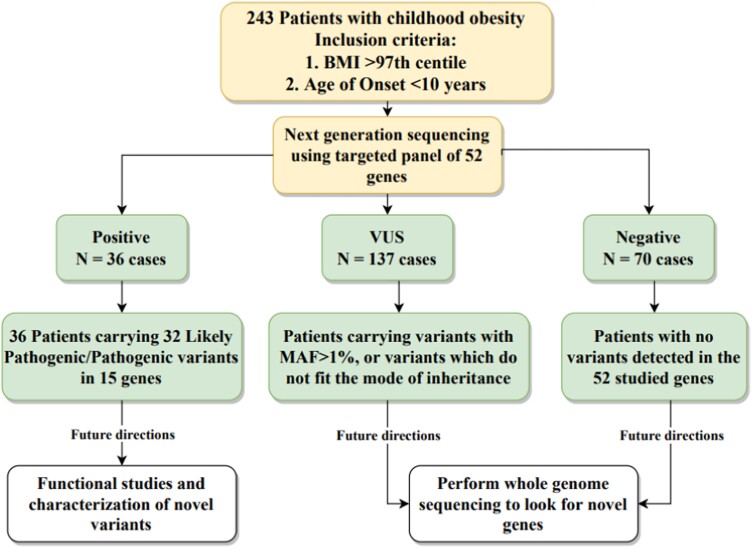
Schematic workflow for identification and characterization of patients. Positive cases (36/243) carry variants that contribute to the molecular etiology of obesity. VUS, variants of uncertain significance; MAF, minor allele frequency.

Our study included 243 probands with severe early-onset obesity including 138 males and 106 females. As per our inclusion criteria, the age of onset was below 10 years; and two-thirds of the probands (n = 163, 68%) were below the age of 5 years. We classified the result into 3 groups based on the genetic findings (Fig. 2): (1) positive cases, these are patients who carry variants predicted to have a deleterious effect on the encoded protein and when the mode of inheritance fitted with what is expected for the specific monogenic obesity form, therefore, they seem to explain the disease in those patients (n = 36); (2) variants of uncertain significance, patients with variants of unclear function (n = 137 cases); and (3) negative cases, these are patients with no variant detected in the 52 studied genes (n = 70 cases).

**Figure 3.**
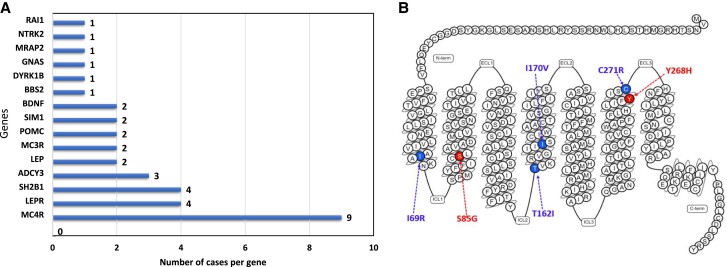
Identification of rare deleterious variants. (A) Cases with a potentially causative variant in 15 genes; 9 probands carried pathogenic variants in *MC4R*. (B) Snake plot of the human *MC4R* gene; novel variants are colored orange; previously reported variants are colored blue. Snake plot modified from GPCRdb www.gpcrdb.org.

We identified 30 rare variants likely associated with obesity in 15 candidate genes (*LEP*, *LEPR*, *POMC*, *MC3R*, *MC4R*, *MRAP2*, *SH2B1*, *BDNF*, *NTRK2*, *DYRK1B*, *SIM1*, *GNAS*, *ADCY3*, *RAI1*, and *BBS2*) (Fig. 3A).

### Melanocortin 4 Receptor

The melanocortin 4 receptor (*MC4R*) gene variants were the most common cause of monogenic obesity in our cohort. We identified 6 rare (minor allele frequency <0.01%) nonsynonymous *MC4R* variants in 10 probands; out of these 6 variants, 2 variants (a compound heterozygous c.253A>G p.S85G and c.802T>C p.Y268H) were novel, while the other 4 (a homozygous c.206T>G p.I69R, 4 heterozygous and 1 homozygous c.485C>T p.T162I, a heterozygous c.508A>G p.I170V, and a heterozygous c.811T>C p.C271R) were reported previously in the literature (Fig. 3B). The variant c.485C>T p.T162I was the most common variant detected in 5 obese children (4 heterozygous and 1 homozygous) with an average BMI of 39 kg/m^2^ (Table 2). We also identified a previously reported variant, c.206T>G, p. Ile69Arg in *MC4R* in 2 severely obese Omani siblings in a homozygous state who were born to consanguineous parents. The siblings, aged 7 and 5 years, had severe obesity and hyperphagia, started to gain weight around the age of 3 years, and at the time of recruitment their weights were 81 and 42 kg, respectively. Their parents, who were heterozygous for the same variant, were severely obese and underwent sleeve surgery. In addition to obesity most of the *MC4R* patients were noticed to have a tall stature (>95th height percentile).

### Leptin

We identified 2 homozygous likely pathogenic variants in the leptin gene (*LEP*) (c.-29+1G>A splice region variant) in 2 morbidly obese siblings; the variant was found in 2 severely obese Egyptian sisters (BMI: 48.4 kg/m^2^ and 44.2 kg/m^2^). The patients were born to first-degree consanguineous heterozygous parents who seem clinically normal. Both patients presented with severely obesity, hyperphagia, obstructive sleep apnea, and fatty liver. This splice variant is predicted to disrupt the splice site of *LEP* and affect the function of *LEP*, and according the ACMG variant interpretation, the variant is classified as pathogenic.

### Leptin Receptor

We identified 3 rare variants in the leptin receptor (*LEPR*) gene, a de novo heterozygous missense, c.2882G>C, p.S961T in a 15-year-old Palestinian patient who started to gain weight from the age of 4; his current weight is 112 kg, with BMI of 33 kg/m^2^. The patient presented with insulin resistance and fatty parenchyma of the pancreas. Additionally, we identified a compound heterozygous variant of uncertain significance (c.2477A>G p.N826S, and c.2705A>G p.H902R) in 4 Qatari probands who were descendants of closely related Arab tribes. These compound heterozygous variants were absent from public population databases (gnomAD, 1000G, and dbSNP) and were predicted to have a deleterious impact on the LEPR protein using some in silico prediction tools (SIFT, Polyphen-2, and CADD).

### Proopiomelanocortin

We identified 2 patients with rare, novel variants in the proopiomelanocortin (*POMC*) gene: c.226A>C p.K76N and c.244T>G p.F82V. The variant c.226A>C p.K76N was found in a 5-year-old severely obese Qatari with a BMI above the 99% percentile, whereas the second variant, c.244T>G p.F82V, was identified in a male Indonesian adolescent with morbid obesity (122 kg at the age of 14 years, BMI: 54 kg/m^2^) with hyperphagia, insulin resistance, and acanthosis nigricans on the neck.

### Src Homology 2B Adapter Protein

Five novel variants in the Src homology 2B adapter protein gene (*SH2B1*) were identified in 3 cases: 3 heterozygous variants (c.1919C>T p. P640L, c.2266G>A p.V756M, and c.2267T>C p.V756A) and 1 compound heterozygous (c.65C>A p.P22Q and c.806G>T p.G269V). The heterozygous variant, c.1919C>T p.P640L, was found in a 17-year-old male patient (145 kg BMI: 59.3 kg/m^2^) who presented with severe obesity, hepatomegaly, and splenomegaly. The proband with compound heterozygous, c.65C>A p.P22Q and c.806G>T p.G269V, was a 7-year-old female patient who started to gain weight from the age of 1 year and weighed 48 kg at the age of 5 years (BMI: 30 kg/m^2^, >99th percentile); both variants are predicted to be deleterious according to several in silico prediction tools.

### Brain Derived Neurotrophic Factor

We detected 2 novel heterozygous variants of the brain derived neurotrophic factor gene (*BDNF*) (c.893G>A p.R298Q and c.596T>C p.L199P) in a 6-year-old boy and a 3-year-old boy with a BMI of 34 kg/m^2^ and 31 kg/m^2^, respectively. The patient with the variant p.R298Q had hepatomegaly, hyperactivity, and hyperphagia, while the boy with p.L199P was hyperphagic, otherwise he had no other endocrinological abnormalities. Both variants lie in a highly conserved region of the *BDNF* gene across different species and are predicted to be disease causing. Additionally, we found a novel heterozygous variant in the BDNF receptor gene, *NTRK2* gene c.1726C>T p.L576F, in a 4-year-old Qatari boy with obesity, hyperphagia, acanthosis nigricans, and obstructive sleep apnea. The p.L576F variant is located in the tyrosine kinase domain of *NTRK2* and is predicted to have a deleterious effect on the encoded protein.

### Adenylate Cyclase 3

We identified 2 novel variants in the adenylate cyclase 3 gene (*ADCY3*): a homozygous frameshift variant, c.2520C>G p.Thr840X, in a 4-year-old girl born to a consanguineous Pakistani parents who presented with severe obesity and signs of insulin resistance. This nonsense variant is predicted to lead to frameshift and premature stop codon. The variant is predicted pathogenic variant according to several in silico prediction tools and ACMG variant classifications. The second novel heterozygous variant c.1222G>A p.G408R was identified in 2 unrelated probands, a Qatari (BMI of 67 kg/m^2^) and an Egyptian patient (BMI of 44 kg/m^2^); both probands are severely obese (BMI >99th percentile). The proband with Qatari origin presented with fatty liver, fatty pancreas, elevated glucose levels (prediabetes), and severe acanthosis nigricans on the neck.

### Melanocortin 3 Receptor

Two heterozygous melanocortin 3 receptor gene (*MC3R*) variants, c.209A>G p.H70R and c.643C>T p.R215T, were found, both of which are predicted to have a deleterious effect on *MC3R* function. A 12-year-old Qatari boy, who started to gain weight from the age of 8 years (current weight: 110 kg, BMI: 36.7 kg/m^2^) was heterozygous for this rare, novel missense variant, p.H70R. Segregation analysis showed that the patient had inherited the variant from his heterozygous father, who was also obese and underwent bariatric surgery. The second *MC3R* variant, c.643C>T p.R215W was identified in a 5-year-old Sudanese girl who started to gain weight at 1 year of age; this patient had hyperphagia, obstructive sleep apnea, and fatty infiltration of the liver.

### Single-Minded Homolog 1

Two heterozygous variants in the single-minded homolog 1 gene (*SIM1*) were found in 2 severely obese patients. This includes a pathogenic frameshift variant (c.428_434del p.H143Pfs*18) in an 11-year-old patient who started to gain weight at the age of 2 years and currently weighs 108 kg (BMI: 48 kg/m^2^). The patient also presented with hepatomegaly, hepatic steatosis, high blood glucose, and dysmorphic features (elongated face, hypertelorism, and brachydactyly). The second variant is a missense change c.1988C>T p.S663L in a 17-year-old adolescent with a birth weight of 3.5 kg who started to gain weight at the age of 10 years; and current weight is 99.6 kg with BMI of 35.4 kg/m^2^.

### Dual-Specificity Tyrosine Phosphorylation Regulated Kinase 1B

We detected a novel missense variant of the dual-specificity tyrosine phosphorylation regulated kinase lB gene *(DYRK1B)*, c.1522C>T p.P508S, in a 4-year-old Bangladeshi obese male with BMI of 35 kg/m^2^ with type 2 diabetes, hypertension, fatty liver with hepatomegaly, and signs of insulin resistance.

### Guanine Nucleotide Binding Protein Alpha Stimulating

In Patient 37 (P37), a 2-year-old Indian boy, whose weight at the time of recruitment was 23 kg and BMI percentile >99th centile, we detected a missense variant c.682C>T p.R228C in the guanine nucleotide binding protein alpha stimulating gene (*GNAS*). The proband inherited the variant from his mother, with BMI of 29.8 but we were not able to examine the mother clinically to check if she has *GNAS*-related features. The variant is predicted to be deleterious and not found in gnomAD database.

### Retinoic Acid Induced 1

We identified a novel heterozygous variant of the retinoic acid induced 1 gene *(RAI1)*, c.785G>A p.R262Q, in a 4-year-old Egyptian boy with obesity, hyperphagia, inattentiveness, hyperactivity, and developmental delay. The patient has some overlapping clinical features with Smith–Magenis syndrome such as obesity, hyperphagia, inattentiveness, hyperactivity, and developmental delay but lacks some common features of Smith–Magenis syndrome such as facial and skeletal anomalies which are subtle and evolve with time. At this early age the phenotype–genotype matching is still not clear.

### Bardet–Biedl Syndrome 2

We found a homozygous missense variant in the Bardet–Biedl syndrome 2 gene (*BBS2*), c. 950A>G p. p.Y317C, in a 5-year-old boy, inherited from a heterozygous consanguineous Somali origin parent. The patient had some common BBS features such as morbid obesity, developmental delay, speech delay, polydactyly, and hypothyroidism. In addition to this, we identified 7 heterozygous BBS variants, and these were classified under the variants of uncertain significance as they did not meet the criteria of autosomal recessive mode of inheritance of BBS. The key clinical characteristics of the patients carrying variants which contribute to the molecular etiology of obesity are summarized in Table 3.

**Table 3.** In silico prediction scores of the variants identified in our cohort

Case	Gene	Variant	ACMG classification	CADD score	SIFT	Polyphen-2	MutationTaster	gnomAD allele frequency
P1	*MC4R*	c.811T>C p.C271R	PM1/PM2/PP3/PP5 Pathogenic	28.2	Deleterious	Probably damaging	Disease causing	0.000004
P2	*MC4R*	c.508A>G p.I170V	PM1/PM2/PP3/PP5/BS2 Pathogenic	22.4	Tolerated	Possibly Damaging	Disease causing	0.00009
P3	*MC4R*	c.253A>G, p.S85G c.802T>C p.Y268H	PM1/PM2/PP3 Uncertain significance (Warm) PM1/PM2 Uncertain significance (Warm)	24.2, 24.2	Deleterious, Deleterious	Probably damaging, Probably damaging	Disease causing, Disease causing	0, 0
P4/P5/P6/P7/P8	*MC4R*	c.485C>T p.T162I	PM1/PM2/PP3 Pathogenic	27.3	Deleterious	Probably damaging	Disease causing	0
P9/P10	*MC4R*	c.206T>G*^a^* p.I69R	PM1/PM2/PM5/PP3 Pathogenic	26.1	Deleterious	Probably damaging	Disease causing	0.000004
P11	*LEPR*	c.2882G>C p.S961T	PM2 Uncertain significance (Cool)	22.5	Deleterious	Probably damaging	Polymorphism	0.000014
P12/P13/P14/P/15	*LEPR*	c.2477A>G p.N826S c.2705A>G P.H902R	PM2/BP4 Uncertain significance (Ice cold) PM2/BP4 Uncertain significance (Cool)	21.2, 24.1	Deleterious Deleterious	Possibly Damaging Probably damaging	Polymorphism Disease causing	0 0
P16/P17	*LEP*	c.-29+1G>A*^a^* p.?	PVS1 Pathogenic	33	NA	NA	Disease causing	0
P18	*POMC*	c.244T>G p.F82V	PM1/PM2/PP3/BP1/BP4 Uncertain significance (Hot)	24.2	Deleterious	Benign	Disease causing	0.00003
P19	*POMC*	c.226A>C p.K76Q	PM1/PM2/PP3/BP4 Uncertain significance (Hot)	23.7	Deleterious	Benign	Disease causing	0
P20/P21	*ADCY3*	c.1222G>A p.G408R	PM1/BP4 Uncertain significance (Cool)	23.7	Benign	Possibly damaging	Polymorphism	0.0001
P22	*ADCY3*	c.2520C>G*^a^* p.T840X	PM2/PP3/PVS1 Pathogenic	47	NA	NA	NA	0
P23	*MRAP2*	c.470A>G p.E157G	PM2 Uncertain significance (Cool)	24.2	Deleterious	Benign	Disease causing	0.000004
P24	*SIM1*	c.1988C>T p.S663L	PM2/BP4 Uncertain significance (Cool)	24.5	Deleterious	Benign	Disease causing	0.000004
P25	*SIM1*	c.428_434del p.H143Pfs*18	PVS1 Pathogenic	NA	NA	NA	NA	0
P26	*NTRK2*	c.1726C>T p.L576F	PM1/PM2/PP3/BP4 Uncertain significance (Hot)	27.7	Deleterious	Probably damaging	Disease causing	0.00004
P27	*BDNF*	c.893G>A p.R298Q	PM1/PM2/PP3/BP1 Uncertain significance (Hot)	26	Deleterious	Probably damaging	Disease causing	0
P28	*BDNF*	c.496T>C L199P	PM1/PM2/BP1 Uncertain significance (Hot)	27.3	Tolerated	Possibly Damaging	Disease causing	0. 00004
P29	*SH2B1*	c.1919C>T p.P640L	PM1/PM2/BP4 Uncertain significance (Warm)	23.3	Deleterious	Benign	Disease causing	0
P30	*SH2B1*	c.65C>A p.P22Q, c.806G>A p.G269V	PM1 Uncertain significance (Cool) PM1/PM2 Uncertain Significance (Warm)	19.79, 24.3	Deleterious, Deleterious	Benign, Probably damaging	Polymorphism, Disease causing	0.00006, 0.000007
P31	*SH2B1*	c.2266G>A p.V756M	PM1/PM2/BP4 Uncertain significance (Hot)	14.35	Tolerated	Benign	Polymorphism	0
P32	*SH2B1*	c.2267T>C p.V756A	PM1/PM2 Uncertain significance (Warm)	14.76	Deleterious (low confidence)	Benign	Polymorphism	0.00007
P33	*DYRK1B*	c.1522C>T p.P508S	PM1/PM2 Uncertain significance (Warm)	23.1	Deleterious (low confidence	Benign	Polymorphism	0
P34	*MC3R*	c.209A>G p.H70R	PM1/PM2 Uncertain significance (Warm)	24.8	Deleterious	Probably damaging	Disease causing	0
P35	*MC3R*	c.643C>T p.R215W	PM1/PM2 Uncertain significance (Warm)	25.4	Deleterious	Probably damaging	Disease causing	0.000004
P36	*GNAS*	c.682C>T p.R228C	PM1/PM2 Uncertain significance (Warm)	25.2	Deleterious	Probably damaging	Disease causing	0
P37	*RAI1*	c.785G>A p.R262Q	PM1/PM2/PP3 Uncertain significance (Hot)	23.9	Tolerated	Probably damaging	Disease causing	0.00002
P38	*BBS2*	c.950A>G p.Y317C	PM2/PP3/BP1 Likely Pathogenic	27.1	Deleterious	Possibly Damaging	Disease causing	0

Majority of the variants (35/38) have high CADD scores (CADD Phred >20).

PM1: The variant is located in a mutational hot spot, PM2: Variant is absent from controls in all databases, PP3: Multiple in silico prediction tools support a deleterious effect on the gene, PP5: Variant was reported as pathogenic by reputable source, BS2: Variant observed in a healthy individual (adult), PM5: Novel missense change at a residue, different variant determined pathogenicity, BP4: Multiple in silico computational tools suggest no impact on gene or gene product, PVS1: Loss of function variant, BP1: Truncating variants are known to cause disease in the gene. ^a^Indicates homozygous variant.

## Discussion

The genetics of obesity in the Middle East, where consanguineous marriages are common, remains understudied. We aimed to shed light on the spectrum of monogenic obesity variants present in such understudied regions of the world by sequencing 243 patients with early-onset severe obesity living in Qatar using a gene panel of 52 obesity-related genes. We established that 14.8% of the patients in our cohort carried likely pathogenic/pathogenic variants in 13 of the targeted genes.

*MC4R* variants were the most common cause of monogenic obesity in our cohort. The variant c.485C>T p.T162I was the most common, which was detected in 5 obese children. This variant was previously reported as a causative variant affecting *MC4R* signaling (15). Interestingly, all 5 probands with this pathogenic variant, p.T162I, were of Qatari origin and descendants of the same tribe. Consistent with our study, a recently published study on the Qatar Genome data identified the same variant in 3 severely obese Qatari adults, with an average BMI of 40.4 kg/m^2^ (16). The variant was also reported previously in 4 severely obese patients from Kuwait and United Arab Emirates (17). The observation of this variant only in Arab patients could provide a plausible explanation that this variant could be a founder variant in the Arabian Peninsula. We further compared the BMIs of the patients carrying homozygous and heterozygous c.485C>T p.T162I *MC4R* variants; the sole patient who was homozygous for the c.485C>T p.T162I variant was more severely obese (17 years, 188 kg, BMI: 69 kg/m^2^) compared with patients who were heterozygous for the same variant (average BMI was 30.1 kg/m^2^) (Table 3). This result is in accordance with previous studies which showed that individuals who were homozygous or compound heterozygous for pathogenic variants were more severely affected than heterozygous carriers (18-20).

Sequence analysis of *MC3R* revealed 2 heterozygous deleterious variants p.H70R and p.R215T in 2 cases in our cohort. Even though there is no clear evidence that heterozygous variants in *MC3R* cause severe monogenic obesity, there are some isolated cases which reported that heterozygous *MC3R* variants are associated to severe obesity; for instance, Lee et al reported a heterozygous missense *MC3R* variant paternally inherited in a severely obese patient and her father with early-onset obesity at the age of 5 years (21). Recently, another study reported 2 heterozygous missense variants (A33D and A259T) in 2 obese individuals; the functional studies of the variants showed defects in cAMP signaling, and a defect in constitutive activation of ERK1/2 signaling that likely led to morbid obesity of the cases (22).

Monogenic forms of obesity due to deficiencies in leptin and leptin receptor genes are characterized by early-onset of rapid weight gain, hyperphagia and insulin resistance, frequent infection, and pituitary hormone deficiency (23). Although, *LEP* and *LEPR* follow an autosomal recessive mode of inheritance, there is a growing evidence that some variants in the heterozygous state in monogenic obesity genes such as *LEP* and *LEPR* could lead to severe childhood obesity, hyperphagia, and pituitary dysfunction (24-26). The 2 extremely rare, novel *LEPR* variants (p.H902R and p.961T), identified in 4 families in the heterozygous state, were located at the intracellular motif of the gene that plays important role in activation the JAK/STAT signaling and leptin binding (27). Further functional work and segregation studies are required to study to potential causality of these rare variants which could be population-specific variants, as they were absent in the public databases.

The hypothalamic neuronal differentiation is controlled by several genes including *BDNF* and *NTRK2*, which regulate the leptin-mediated synaptic plasticity of hypothalamic neuros, and *SIM1*, a transcription factor essential for the development of neurons of the paraventricular nucleus (28). Interestingly, pathogenic variants in these genes leads to severe childhood obesity commonly through autosomal dominant mode of inheritance, and some heterozygous variants in these genes have been described to cause delayed development in addition to obesity (29, 30). Intellectual ability and developmental milestones of the patients with variants on these genes in our cohort were normal except for the proband with *BDNF* p.R298Q variant, who showed autistic behavior and hyperactivity.

Unlike the well-established autosomal mode of inheritance in nonsyndromic obesity, there are only a few studies that have investigated the implication of heterozygous variants on obesity in the large scale (26, 31). These findings concluded that heterozygous variants resemble a phenotype similar to those patients who are homozygotes for the variants of interest. Consistent with the aforementioned results, 1 of the common features we observed in our study based on phenotype–genotype correlation is that heterozygous variants in the leptin–melanocortin pathway could lead to childhood obesity.

The proband with the homozygous nonsense *ADCY3* variant in our cohort has a Pakistani origin, and the highest prevalence of obesity due to *ADCY3* in a single cohort was observed in Pakistan. *ADCY3* knockout mice are obese, whereas *ADCY3* gain of function mutation protects mice against obesity. In addition to obesity, loss of function mutations in *ADCY3* cause anosmia in both mice and humans; unfortunately we did not perform a smell test in our patient.

*DYRK1B* is associated with a metabolic syndrome known as abdominal obesity-metabolic syndrome 3 (AOMS3), which is characterized by abdominal obesity, type-2 diabetes, hypertension, and heart diseases (OMIM #615812). *DYRK1B* inhibits the sonic hedgehog pathway, which leads to increased adipogenesis. The gain of function of *DYRK1B* also upregulates the expression of glucose-6-phosphatase, a glucogenic enzyme that regulates glucose production (32). The heterozygous variant identified in this study, c.1522C>T p. Pro508Ser seems likely to be a benign variant based on in silico prediction tools; SIFT predicted the variant to be deleterious with low confidence; MutationTaster predicted the variant as polymorphism with a summary of amino acid changed: protein features might be changed and splice site changes. However, based on the genotype–phenotype association, the patient in addition to obesity has type 2 diabetes, hypertension, fatty liver with hepatomegaly, and signs of insulin resistance, which are consistent with the previously described AOMS3 (OMIM:615812), which is caused by variants on *DYRK1B*.

Although it is difficult to estimate the prevalence of monogenic obesity in Qatar based on our study due to our small sample size, it seems that the prevalence is higher than that reported in European, Turkish, and Guadalupian populations (3, 7, 8). The identification of molecular causes of obesity plays an important role in personalized therapeutic approaches for patients with monogenic obesity. Previously, the therapeutic approach for monogenic obesity, especially for patients with the congenital leptin deficiency was limited to metreleptin replacement therapy (33). Metreleptin is a 147 amino acid chain that differs from the native leptin protein by an extra methionine residue at the amino terminus. Recently, novel drugs, such as setmelanotide, have been approved by the FDA that could be used for weight management for some forms of monogenic obesity (34). Setmelanotide, a MC4R agonist mimics the POMC-derived alpha melanocyte-stimulating hormone and helps in weight loss, increased energy expenditure, increased insulin sensitivity, and decreased hyperphagia in patients with genetic defect in genes such as *POMC*, *MC4R*, *LEPR*, *PCSK1*, and *MAGEL2* (35).

Liraglutide, a glucagon-like peptide receptor agonist (GLP1-RA), shares high amino acid sequence homology with GLP-1, was first approved for type 2 diabetes and later approved for treatment of common obesity; it reduces appetite and leads to weight loss in obesity caused by *MC4R* pathogenic variants. These drugs have been used to treat patients with monogenic obesity caused by pathogenic variants in the leptin–melanocortin pathway (36, 37). Therefore, most of the molecular diagnosis of our patients is of a great importance for improved clinical management with these recently approved personalized obesity drugs, and also of great importance for improving clinical management and family care strategies.

The genetic cause of most of the cases in our cohort (86%) remained unexplained (negative cases or cases with variants of uncertain significance). This could be due to several different reasons. First, even though the targeted panel is 1 of the largest panels to investigate obesity, it is designed to cover the coding regions and flanking regions of the exons, and it is possible that variants in the intronic regions that may contribute to disease pathogenesis through regulatory effects were not covered. Second, we sequenced only probands; however, to have a better understanding of the phenotype–genotype correlations, trio sequencing may have proven more intuitive and allow segregation analysis for looking a novel genetic etiology of the obesity and could have also facilitated the confirming the possible role of some of variants with uncertain significance. Lastly, large structural variants that could play a role in the pathogenicity of obesity were not investigated in our cohort.

### Conclusion

Approximately 14.8% of probands in our cohort carried rare variant/s that can explain their severe early-onset obesity, 23 of which were novel to this population and 7 previously described pathogenic variants, most of which are in the leptin–melanocortin pathway. These results support the importance of the hypothalamic melanocortin pathway in energy homeostasis and development of obesity, and the importance on exploring understudied populations to better understand the landscape of monogenic obesity. The findings from this study emphasize that with the continuing reduction in costs of NGS, screening monogenic obesity genes should be considered routine practice for patients with early-onset obesity. Early diagnosis potentially improves obesity management, early interventions, and helps to identify patients who may benefit from the emerging personalized therapeutic interventions for monogenic obesity.

## Data Availability

Some or all datasets generated during and/or analyzed during the current study are not publicly available but are available from the corresponding author on reasonable request.

## Abbreviations

ACMG: American College of Medical Genetics and Genomics
*ADCY3*: adenylate cyclase 3 gene
*BBS2*: Bardet–Biedl syndrome 2 gene
BDNF: brain derived neurotrophic factor gene
BMI: body mass index
*DYRK1B*: dual-specificity tyrosine phosphorylation regulated kinase lB gene
*GNAS*: guanine nucleotide binding protein alpha stimulating gene
*LEP*: leptin gene
*LEPR*: leptin receptor gene
*MC3R*: melanocortin 3 receptor gene
*MC4R*: melanocortin 4 receptor gene
NGS: next-generation sequencing
PCR: polymerase chain reaction
*POMC*: proopiomelanocortin gene
*SH2B1*: Src homology 2B adapter protein gene
*RAI1*: retinoic acid induced 1 gene
*SIM1*: single-minded homolog 1 gene
SNP: single nucleotide polymorphism
